# A survey of thrombosis experts evaluating practices and opinions regarding venous thromboprophylaxis in patients with active cancer hospitalized with an acute medical illness

**DOI:** 10.1186/s12959-015-0040-6

**Published:** 2015-02-14

**Authors:** Patricia Moretto, Junghyun Park, Marc Rodger, Grégoire Le Gal, Marc Carrier

**Affiliations:** Kaplan Instituto de Oncologia, Porto Alegre, Rio Grande do Sul Brazil; Department of Medicine, The Ottawa Hospital Research Institute, University of Ottawa, 501 Smyth Road, Box 201A, Ottawa, ON Canada; Institut de Recherche de l’Hôpital Montfort, University of Ottawa, Ottawa, Canada

## Abstract

**Background:**

Current clinical practice guidelines recommend the use of prophylactic doses of low molecular weight heparins for cancer patients requiring hospitalization for acute medical illness. However, a recently published meta-analysis suggested that the risk-benefit ratio of current thromboprophylaxis regimens administered to all cancer patients admitted for medical illness is unclear. We sought to assess the clinical equipoise in using thromboprophylaxis for hospitalized medically ill cancer patients.

**Methods:**

An electronic survey was conducted. The target sample included Thrombosis experts and members of Thrombosis Canada or the VECTOR research group.

**Results:**

The survey was distributed 54 participants. The final response rate was 67% (36/54). The majority (75%; 95% CI: 60.3 to 85%) of responders indicated that the benefits of pharmacological parenteral thromboprophylaxis outweigh the risks. However, 63.9% (95% CI: 50.6 to 77.3%) believe that there is still clinical equipoise around the use of thromboprophylaxis in this patient population, and 88.9% (95% CI: 77.3 to 95.8%) would consider participating in a randomized trial—30.6% and 58.3% in a placebo-controlled or comparison of different agents/dosing-controlled randomized trial, respectively. For participants who would consider a randomized-controlled trial comparing different doses of thromboprophylaxis agents, the MCID was 2% between the two arms. The most common drug to be compared was enoxaparin (26%), and the two suggested doses were 30 mg and 40 mg SC twice daily.

**Conclusions:**

Our clinical survey of thrombosis experts confirms that there is equipoise regarding the use of current regimens of parenteral pharmacological thromboprophylaxis in medically ill cancer patients. A majority of physicians would participate in a randomized-controlled trial comparing different dose of LMWH. The MCID in the risk of VTE identified was 2%.

## Introduction

Venous thromboembolism (VTE) which includes both pulmonary embolism (PE) and deep vein thrombosis (DVT) is a frequent complication in cancer patients and one of the leading causes of death in this population [1,2]. The presence of cancer itself and the added risk of hospitalization compound the risk of developing a VTE [3]. Therefore, current clinical practice guidelines from the American Society of Clinical Oncology, National Comprehensive Cancer Network, the American College of Chest Physicians (ACCP) and the European Society of Medical Oncology all recommend the use of usual prophylactic doses of low molecular weight heparins (LMWH) for cancer patients requiring hospitalization for acute medical illness in the absence of bleeding or other contra-indications to anticoagulation [4-7]. These recommendations are based on extrapolation from large randomized controlled trials assessing the efficacy and safety of pharmacological thromboprophylaxis in medically-ill hospitalized patients [8-10]. However, a recently published meta-analysis of the cancer subgroups from these trials suggested that the risk-benefit ratio of current thromboprophylaxis regimens administered to all cancer patients admitted for medical illness is unclear and additional randomized trials are necessary to establish which cancer patients may benefit from the routine administration of LMWH [11]. The principal aim of this survey was to assess the clinical equipoise in using thromboprophylaxis for hospitalized medically ill cancer patients and to evaluate the potential participation in a future randomized controlled trial.

## Methods

An Internet-based survey was conducted using Survey Monkey. The target sample included Thrombosis experts and clinicians actively involved in the management and prevention of thrombosis in adult patients—identified through Thrombosis Canada and VECTOR (VEnous thromboembolism Clinical Trials and Outcomes Research) member rosters. Thrombosis Canada is a Canadian registered non-profit organization with no commercial interests dedicated to furthering education and research in the prevention and treatment of thrombotic vascular disease. Members include medical generalist and specialist that have made significant contributions to the body of knowledge in vascular medicine (http://thrombosiscanada.ca/). The VECTOR group is a large multi-centre collaborative research group including 9 academic centers with a focus on venous thromboembolism clinical trials and studies. An email introducing the survey with the link was initially distributed. Confidentiality was guaranteed and participation in the survey was interpreted as consent. A total of two reminders were also sent at 1-week intervals. Prior to distribution, the survey was reviewed by eight physicians (medical oncology, radiation oncology, and thrombosis) at The Ottawa Hospital to ensure validity and accuracy. The survey was conducted in multiple-choice format. General questions about pharmacological parenteral thromboprophylaxis in cancer patients, use of established guidelines, risk/benefit ratio in this population, and the clinical equipoise around the subject were asked. Information was also collected regarding their willingness in enrolling patients in a clinical trial, schedules, doses, comparators, and minimal clinically important difference (MCID).

The results of the survey are stated using descriptive data (percentages and 95% CI). Analytic comparisons were made among relevant demographic subgroups regarding practice patterns and willingness to conduct future trials of thromboprophylaxis in hospitalized cancer patients. Analyses were conducted using Stats Direct software (version 2.7.9)

## Results

The survey was distributed via email to 54 participants. Following the initial email, two reminders over 1-week intervals were sent and the final response rate was 67% (36/54). The final sample consisted of hematologists (68.6%), internists (25.7%), and medical oncologists (5.7%). Baseline characteristics of the respondents are depicted in Table 1. Of the participants, 57.1% were male and 60% had been practicing for more than 10 years. All respondents practiced in academic centers, and though 54.3% spent less than 25% of their practice working with cancer related VTE, 17% of the participants reported more than 50%.

**Table 1:** **Baseline characteristics of the respondents**

	**%**
Hematologists	69%
Internists	26%
Medical oncologists	6%
Male	57%
Practicing for more than 10 years	60%
Practice in academic center	100%
Spent more than 50% of practice working with cancer related VTE	17%

Given the scenario of an adult patient with active cancer (non-hematological malignancy) hospitalized for acute medical illness (not due to surgery), who is neither actively bleeding nor at high risk of bleeding, the majority of physicians (75%; 95% CI: 60.3 to 85%) would always recommend the use of thromboprophylaxis. For this group of patients, the majority (75%; 95% CI: 60.3-85%) of responders indicated that the benefits of pharmacological parenteral thromboprophylaxis outweigh the risks. However, 63.9% (95% CI: 50.6 to 77.3%) believe that there is still clinical equipoise around the use of thromboprophylaxis in this particular patient population, and 88.9% (95% CI: 77.3 to 95.8%) would consider participating in a randomized trial—30.6% and 58.3% in a placebo-controlled or comparison of different agents/dosing-controlled randomized trial, respectively. For those who would consider a placebo-controlled trial, the absolute reduction in symptomatic VTE reported as the MCID was 2% (63.6%) and the absolute “acceptable” increase in major bleeding events was 1% (63.6%). For participants who would consider a randomized-controlled trial comparing different doses of thromboprophylaxis agents, the MCID was 2% between the two arms. The most common drug to be compared was enoxaparin (26%), and the two suggested doses were 30 mg and 40 mg SC twice daily.

## Discussion

Our clinical survey of thrombosis experts confirms that there is equipoise regarding the use of current regimens of parenteral pharmacological thromboprophylaxis in medically ill cancer patients. A majority of physicians would participate in a randomized-controlled trial comparing different dose of LMWH. The MCID in the risk of VTE identified was 2%.

As expected, the majority (75%) of participants recommend thromboprophylaxis in adult patients with cancer hospitalized for acute medical illness, and reported that the risk of parenteral pharmacological thromboprophylaxis outweighs the risk of bleeding in these patients. The ACCP guidelines were the most frequently (87.9%) selected guidelines. The lack of evidence from randomized-controlled trials supporting the efficacy of thromboprophylaxis is generating significant clinical equipoise centered on the dosing of parenteral pharmacological thromboprophylaxis in this patient population.

A majority of clinicians (68%) selected LMWH as their preferred pharmacological thromboprophylactic agent. This is not surprising given that LMWH have a better safety profile compared to unfractionated heparin. Unfractionated heparin (UFH) is easier to use, is associated with higher risk of heparin-induced thrombocytopenia (1% for UFH compared to 0.1% for LMWH) and is associated with lower risk of a major bleeding among hospitalized medically ill patients [12]. Similarly, LMWH was preferred over fondaparinux or the direct oral Factor Xa inhibitors (rivaroxaban or apixaban). Post-hoc analyses from randomized-controlled trials comparing fondaparinux or rivaroxaban with LMWH suggest that specific factor Xa inhibition might be less efficacious than LMWH inhibition in cancer patients [13,14]. The MAGELLAN trial assessed the efficacy and safety of rivaroxaban in medically ill patients. The supplemental data showed that patients with active cancer randomized to 35 ± 4 days of rivaroxaban had more asymptomatic proximal or symptomatic VTE than patients receiving only 10 ± 4 days of enoxaparin (9.9% (20/202) *vs.* 7.4% (15/203)) [13]. Although this difference was not statistically significant due to the small number of cancer patients enrolled, there was a statistically significant increase in major and clinically relevant non-major bleeding episodes with rivaroxaban compared with enoxaparin (5.4% *vs.* 1.7%). Therefore, parenteral pharmacological thromboprophylaxis using LMWH seems to be the optimal choice.

When considering a randomized-controlled trial to determine the optimal dose of LMWH, most of the participants favoured a trial assessing different doses of LMWH. Enoxaparin has been shown to decrease D-dimer levels and prothrombin fragments (F1 + 2) in hospitalized medically ill patients [15,16]. Currently, a dose of 40 mg would be the recommended dose in these patients. Higher doses of enoxaparin (80 mg) have been reported to significantly decrease the peak thrombin levels compared to the lower dose (40 mg) in hospitalized cancer patients, suggesting that the pro-thrombotic state related to cancer might be attenuated by higher doses of LMWH [16]. Previous clinical studies in other high-risk cancer populations assessing the use of higher doses of LMWH have also demonstrated significant benefits without increasing the risk of bleeding [17-19]. Therefore, assessing different doses of LMWH has a strong biological and clinical rational.

It is important to acknowledge the limitations of our survey. The target sample size was relatively small which might introduce selection bias. However, the response rate was high and it is important to emphasize that the survey targeted thrombosis experts only in order to provide the most relevant opinions regarding the assessment of the MCID. In addition, the sample size was limited to Canadian clinicians from academic centers, and therefore, may not reflect the worldwide opinion on the topic.

In conclusion, our survey shows that there is clinical equipoise regarding the use of pharmacological thromboprophylaxis in medically ill cancer patients, and calls for trials comparing different doses of thromboprophylaxis in these patients.
